# Longitudinal White Matter Maturation in Preterm Infants: Functional Pathway-Specific Trajectories and Associations with Motor Outcomes

**DOI:** 10.3390/jcm15020823

**Published:** 2026-01-20

**Authors:** Gang Yi Lee, Yong Hun Jang, Joo Young Lee, Hyuna Kim, Bong Gun Lee, Mi Jung Kim, Hyun Ju Lee

**Affiliations:** 1 Department of Translational Medicine, Hanyang University Graduate School of Biomedical Science and Engineering, Seoul 04763, Republic of Korea; lgl6870@naver.com; 2 Department of Pediatrics, Hanyang University College of Medicine, Seoul 04763, Republic of Korea; ryanjang93@naver.com (Y.H.J.); ks1253@naver.com (J.Y.L.); ruykaz1@hanmail.net (H.K.); 3 Department of Orthopedic Surgery, Hanyang University College of Medicine, Seoul 04763, Republic of Korea; bglee@hanyang.ac.kr; 4 Department of Rehabilitation Medicine, Hanyang University College of Medicine, Seoul 04763, Republic of Korea; kimmjreh@hanyang.ac.kr; 5 Division of Neonatology and Development Medicine, Hanyang University Hospital, Seoul 04763, Republic of Korea; 6 Hanyang Institute of Bioscience and Biotechnology, Hanyang University, Seoul 04763, Republic of Korea

**Keywords:** preterm infants, white matter, trajectory, diffusion tensor imaging

## Abstract

**Background:** During the first 2 years of life, human white matter (WM) undergoes rapid development, establishing a structural foundation for later neurodevelopment. **Methods:** We conducted a mixed-model analysis for repeated measures to investigate the developmental trajectories of functionally distinct 26 WM pathways between preterm and full-term groups during the first 2 years of life using diffusion tensor imaging (total scans = 174; preterm = 58; full-term = 23). **Results:** We observed significant differences between the preterm and full-term groups in the developmental trajectories associated with motor function (left corticospinal tract and left pre-primary motor cortex connection tracts), visual processing (bilateral pathway between the V1/V2 and V4, PV-MT, pathway connecting the V1/V2 and V5/MT, and optic radiation), and cognition (genu, body, and splenium of the corpus callosum). Furthermore, inter-regional correlation matrix analysis revealed stronger connectivity, specifically within motor- and visual-related pathways, in the preterm group than that for the full-term group, suggesting an adaptive mechanism that supports circuit-level resilience following preterm birth. Moreover, in the model investigating the associations between the WM individual rate of change and long-term neurodevelopmental outcomes, the middle cerebellar peduncle (MCP) tract showed the strongest associations with motor scores, suggesting that faster maturation of the MCP tract may enhance motor functions as a key compensatory mechanism following preterm birth. **Conclusions:** Delineating the longitudinal change rates of specific WM pathways not only deepens our understanding of the neurodevelopmental sequelae of prematurity but also highlights their potential as early biomarkers to guide timely interventions.

## 1. Introduction

Brain development in the first 2 years of life is the most dynamic and profound process [1,2]. During this time, studies have shown that the brain size reaches 80–90% of the adult volume by age 2 years [3], the cortex, including the cortical and subcortical growth, expands rapidly [1,4,5], and the rate of brain white matter (WM) myelination peaks [6]. In particular, during WM development, as axonal fibers appear to be more directionally organized and fiber myelination occurs, diffusion anisotropy is high with reduced isotropic diffusion [7,8].

Converging evidence suggests that the neuroanatomical structure of WM is intrinsically linked to changes in brain global efficiency, which sets the stage for subsequent neurodevelopment [9,10,11]. Given that the onset of axonal organization and WM myelination coincides with the period of preterm birth, the susceptibility and vulnerability of WM maturation to extrauterine environmental perturbations raise concerns about atypical brain development and neurodevelopmental deficits [12,13,14]. Notably, compared with term-born infants, survivors of preterm birth have higher rates of motor, cognitive, and language impairments, and are likely to undergo altered maturation and impaired growth of specific WM pathways. Many recent studies support this contention, with low axonal fiber density and delayed myelination in the preterm group over the first 2 years [15,16,17,18].

A normal trajectory of the early neonatal maturation of WM bundles follows a nonlinear trajectory, with maturation rates varying across brain regions. Projection tracts, including motor and sensory tracts, show a higher maturation degree at birth, while association tracts, such as higher-order associative areas, continuously show a lower maturation degree in the first 2 years [19,20,21]. In particular, previous neonatal diffusion tensor imaging (DTI) studies have shown that maturation of the corpus callosum and corticospinal WM proceeds rapidly in neonatal brains after birth [2]. Early maturing tracts may be experience-dependent and foster efficient and coordinated transmission of signals across the entire brain, laying the groundwork for neural maturation. The rate of WM development during this window underlies a higher degree of plasticity and experience-dependent shaping of neural circuits, which serve as key mechanisms for adaptation to environmental changes and recovery from injury. In the developing brain, the heightened sensitivity and plasticity of neural tissue may render preterm infants more vulnerable to adverse events such as inflammation, hypoxia, and ischemia. In contrast, plasticity also holds the potential to serve as a neuroprotective mechanism, whereby enriching early experiences may promote the normalization of neural development. This heightened plasticity may support more effective recovery processes, highlighting the possibility of enhancing developmental WM pathways and improving neurobehavioral outcomes in preterm infants [22,23,24].

Studies exploring the trajectories of WM microstructure development have been conducted in typically developing children during the critical period from birth to 2 years of age. In addition, many existing research is cross-sectional, and the lack of repeated measures within the same infants limits our understanding individual differences in preterm infants. Despite notable interindividual variability, there remains a limited understanding of how the brain changes during development and its associated neurobehavioral development. Along these lines, a longitudinal study enables the tracking of intra-individual changes over time and allows us to definitively establish the causality or temporal dynamics of WM development. Therefore, there is a need for longitudinal studies using advanced analysis methods for longitudinal data to expand our knowledge of the effects of preterm birth on WM development. In light of this need, the present study corroborated the microstructural maturation of functionally segregated WM tracts in a longitudinally scanned cohort of preterm and full-term infants during the first 2 years of life. First, we estimated the trajectories and inter-tract relationships of each diffusion metric in functionally related pathways over the first 2 years of life using linear mixed effect (LME) models between the preterm and full-term groups. Second, we explored the associations between individual longitudinal changes in the regional WM microstructure and neurodevelopmental outcomes, identifying the foundational basis of early pathway maturation for later functional development.

## 2. Materials and Methods

### 2.1. Study Populations

**Figure 1:**
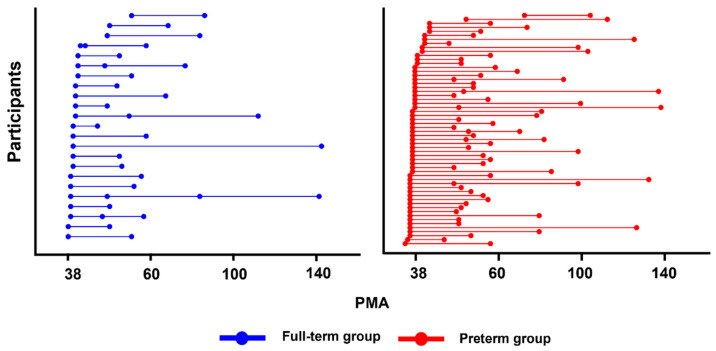
Age at magnetic resonance imaging scans for participants (preterm group shown in red, full-term group shown in blue). Each of the scans (122 preterm group scans, 52 full-term group scans) are represented by a circle, while each of the participants (57 PT, 23 full-term) has a unique row with their scans connected by a line.

This study reports a longitudinal cohort of 81 infants from birth to 2 years of age (58 preterm and 23 full-term infants) (Figure 1). All fifty-eight preterm group born at <37 weeks of gestational age (GA) and admitted to the neonatal intensive care unit of Hanyang University Hospital were eligible for inclusion in the preterm cohort of the Hanyang Inclusive Clinic for Developmental Disorders between 2018 and 2023. Twenty-three full-term infants were recruited from the Hanyang University Hospital Newborn nursery within 2 years of age between 2018 and 2023. All infants, including the preterm and full-term groups, were included in the analysis if they had valid DTI tract data from at least two time points between the term-equivalent age (TEA; that is, predicted due date based on a term pregnancy of 42 weeks) and 26 months of age. From the eligible 58 preterm and 23 full-term infants, 122 and 52 scans were obtained, respectively. The initial scans were conducted at TEA (53 preterm scans, 19 full-term scans), with subsequent scans scheduled based on age-dependent intervals. Follow-up scans were conducted at either 2–6 months (33 scans of preterm, 20 scans of full-term), or 7–12 months (20 scans of preterm; 7 scans of full-term); for children aged over 1 year, additional scans were conducted at 13–18 months (10 scans of preterm; 3 scans of full-term), or 19–26 months (6 preterm group, 3 full-term group). Demographic information is summarized in Figure 1 and Table 1.

The exclusion criteria included brain abnormalities, intraventricular hemorrhage, periventricular leukomalacia, and neuropsychiatric disorders. The Hanyang University Hospital Institutional Review Board approved the present study protocol, and informed consent for participation in the study was obtained from the infants’ parents. All procedures were performed in compliance with the principles of the Declaration of Helsinki.

### 2.2. Developmental Assessment

Eligible follow-up cohorts (*N* = 81) underwent neurodevelopmental tests at 4–26 months of age were enrolled. We analyzed long-term neurodevelopmental outcomes using the Third Edition of the Bayley Scales of Infant and Toddler Development (BSID-III). These standardized assessments provide four subscale scores (cognitive, language, motor, and social emotion). The BSID-III tests yielded standardized scores with a mean of 100 and a standard deviation of 15.

### 2.3. Magnetic Resonance Imaging (MRI) Acquisitions

Individuals were scanned using a whole-body 3T MRI scan (Philips, Achieva, 16-channel phase-array head coil; Best, The Netherlands) without sedation. During the MRI scans, an experienced pediatrician monitored the heart and respiration rates of each infant using the pulse oximeter to ensure their safety. DTI data were acquired with a single-shot spin-echo-planar sequence with the following parameters: voxel sizes = 1.56 × 1.56 mm^2^, slice thickness = 2 mm, field of view = 120 × 120 mm, repetition time (TR)  =  4800 ms, flip angle  =  90°, echo time (TE)  =  75 ms, number of electrostatic gradient directions  =  15, number of averages  =  2, b-value  =  800 s/mm^2^, and total acquisition time  =  6 min 17 s. The slices were acquired in an axial orientation aligned parallel to the anterior–posterior commissure line. A total of 40–50 slices encompassed the entire hemisphere and brainstem. Motion artifacts in diffusion MRI were quantified for each participant within each group, including measures of absolute and relative volume-to-volume motion and the percentage of outlier volumes, using the EDDY QC tool [25] in the FMRIB’s Software Library (FSL 6.0.7.13, https://fsl.fmrib.ox.ac.uk/, accessed on 15 September 2024) [26].

### 2.4. Data Preprocessing

A total of 174 DTI scans were processed using FSL [26]. We removed the skull and non-brain tissues from the non-diffusion-weighted image (b0 volume) using a Brain Extraction Tool [27]. The MRI susceptibility-induced fields, motion artifacts, and eddy current distortions were corrected by outlier replacement using an eddy correction tool. To prevent low-frequency intensity inhomogeneity in the b0 image, we applied an N4 bias field correction using Advanced Normalization Tools [28] for bias field estimation. Each voxel in the diffusion-weighted image was estimated using a simple least-squares algorithm. Subsequently, scalar diffusion metrics, such as fractional anisotropy (FA), mean diffusivity (MD), axial diffusivity (AD), and radial diffusivity (RD), were calculated.

### 2.5. DTI Atlas Registration

To investigate the microstructure of the pathway of interest, a pathway atlas was constructed by applying a threshold of 0.1. All the FA images were aligned to the JHU neonatal FA atlas and transformed into the atlas space using a normalization tool. The pathway atlas was projected onto individual FA images using an inverse matrix obtained through linear and nonlinear registration, and the mean diffusion (trace) values of the brain regions with a probability >10% in the DTI tractography-based probability map were measured. The probabilistic map of the WM tracts was overlaid on the JHU Neonatal Atlas to quantify the FA, MD, AD, and RD values of the pathway. DTI measurements were obtained from multiple regions of interest (ROIs) positioned bilaterally within individual WM tracts. Of these, we specifically selected 26 WM tracts as ROIs, which were classified into one of the functional pathways: motor (corticospinal tract [CST]; middle cerebellar peduncle [MCP]; pathway connecting the premotor and primary motor cortices [PPMC]), cognitive (genu, body, and splenium of the corpus callosum [GCC, BCC, and SCC]), visual (pathway between the V1/V2 and V4 [PVV4]; pathway connecting the V1/V2 and V5/MT [PVMT]; optic radiation [OR)), limbic/language (cingulum [CG], uncinate fasciculus [UNC], inferior fronto-occipital fasciculus [IFO], and inferior longitudinal fasciculus [ILF]), and somatosensory (thalamo-primary somatosensory cortex [TPSC] and acoustic radiation [AR]) [29].

### 2.6. Statistical Analysis

All statistical analyses were conducted using R software (version 4.4.2) (https://www.r-project.org/) [30]. Descriptive statistics were computed for all clinical and behavioral variables. Group differences were examined using independent-samples *t*-tests to compare the preterm and full-term groups. To control for multiple comparisons, false discovery rate (FDR) correction was applied.

#### 2.6.1. LME Model Fitting

To examine the relationship between age and groups for each diffusion metric in a longitudinal study, the LME model was used to describe the average measurement and temporal trajectory using a linear regression paradigm in longitudinal study [31]. The LME model was constructed in R using the lme4 package [32]. The model outlined below describes our linear mixed model.

WM metrics = log(PMA) + Sex + GA + Group + log(PMA)x Group + (1 + log(PMA)|Subject ID)

In the fitting model, sex, GA, age, group, and the interaction between age and group were included as fixed effects. In addition, random intercepts and slopes were entered as random effects for repeated observations per participant. A random slope was extracted for each participant as an index of the rate of change for each WM metric. Trajectory plots were generated using the values predicted from the fitted model obtained through the predicted function in R [33]. For multiple comparisons, significant *p*-values were adjusted using FDR for each of the five pathway classes, including motor, cognitive, visual, limbic/language, and somatosensory functions, across the four WM metrics.

#### 2.6.2. Inter-Regional Partial Correlations of WM Residuals from Longitudinal Models

To determine group differences in the association between WM microstructural development in the 26 pathways over the first 2 years of life, inter-regional partial correlation coefficients between each region were calculated. In longitudinal studies with repeated measurements, traditional correlation coefficients such as Pearson’s r may be inappropriate, as repeated measures within the same individual violate the assumption of independence. Furthermore, the observed associations between two variables may be confounded by shared covariates (such as GA, sex, and PMA), rather than reflecting the intrinsic relationship between the variables of interest. To address this issue, we proposed using the LME model to compute the residuals of repeated measurements after adjusting for confounders. These residuals represent the unexplained variability of each measurement after accounting for covariates and individual-level random effects, and can be used to assess the pure association between variables [34]. For each WM tract, we fitted the LME as follows:

WM metrics = log(PMA) + Sex + GA + (1 + log(PMA)|Participant ID)

This model included PMA, sex, and GA as fixed effects, with individual-specific random intercepts and slopes to account for individual developmental trajectories. Moreover, to avoid the risk of model overfitting, which can arise when using the same LME model structure for both residual extraction and correlation estimation, we estimated the correlations under an independent assumption, treating the residuals as independent observations [34]. Finally, inter-regional partial correlations across the 26 regions were compared between the two groups (the full-term and preterm groups), and the differences were visualized as 26 × 26 heatmaps, allowing for the comparison of inter-regional structural synchrony between groups during the first 2 years.

#### 2.6.3. Associations Between Individual WM Developmental Slopes over the First 2 Years and Neurodevelopmental Outcomes in the Preterm Group

Partial correlation analysis was used to identify the association between individual WM developmental changes (i.e., slope) and neurodevelopmental outcomes in the preterm group. First, we estimated the rate of individual change for each tract using the above LME model in R. Subsequently, correlation analysis was performed to examine the associations between these WM slopes and neuropsychological outcomes. The outcome measures included four subdomains of neuropsychological assessments using the BSID III: cognition, language, motor function, and socio-emotional skills. A correlation matrix was computed between the individual WM slopes (X) derived from the LME model and the individual neuropsychological scores (Y), identifying the significant components that captured the strongest correlations.

## 3. Results

### 3.1. Demographic and Clinical Characteristics

A total of 81 participants were included in the study, comprising 58 preterm and 23 full-term infants. The preterm group showed a significantly lower gestational age (*p* < 0.001), age at first scan (*p* = 0.002), birth weight (*p* < 0.001), and proportion of males (*p* = 0.017) than the full-term group. There were no significant group differences in the maternal age (*p* = 0.300). On the BSID III, the preterm group showed significantly lower scores in the language (*p* = 0.042) and motor (*p* = 0.008) than the full-term group. However, there were no significant differences in cognition (*p* = 0.130) or social emotion (*p* = 0.973) scores between the two groups. Detailed descriptive and statistical results are presented in Table 1.

### 3.2. Longitudinal Group by Age Interactions in Diffusion Metrics

Among the 26 WM pathways assessed across the four-diffusion metrics, the preterm group initially exhibited lower AD, MD, and RD values and higher FA values than the full-term group. However, over time, the preterm group demonstrated a steeper decrease in AD, MD, and RD and an increase in FA.

**Figure 2:**
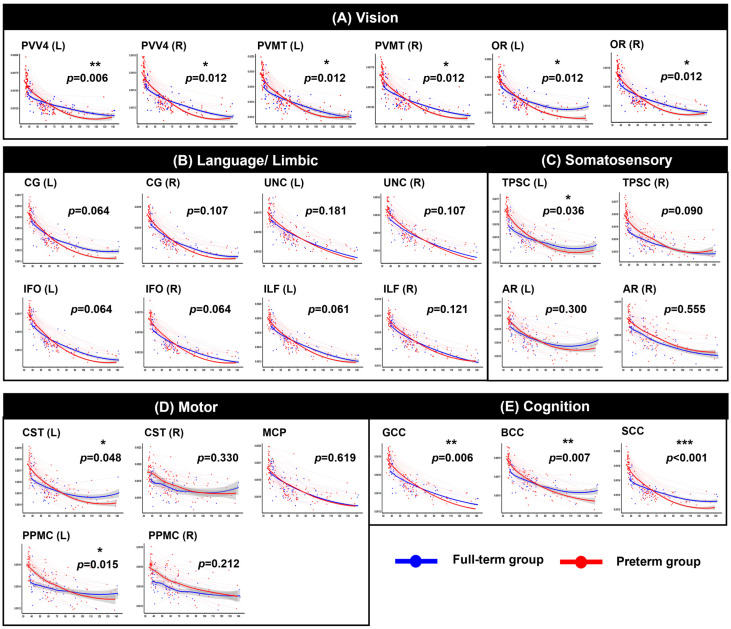
Longitudinal trajectories of axial diffusivity (AD) across PMA. Each circle represents an individual scan, and thin lines connect repeated measures from the same participant. The red color indicates the preterm group, and the blue color indicates the full-term group. The shaded gray area indicates the 95% confidence interval of the fitted curve. Abbreviations: CST, corticospinal tract; PPMC, pathway connecting the premotor and primary motor cortices; MCP, middle cerebellar peduncle; gCC, genu of the corpus callosum; sCC, splenium of the corpus callosum; PV–V4, pathway between the V1 (primary visual area)/V2 (secondary visual area) and V4; PV-MT, pathway connecting the V1/V2 and V5/MT (middle temporal visual area); OR, optic radiation; CG, cingulum; UNC, uncinate process; IFO, inferior fronto-occipital fasciculus; ILF, inferior longitudinal fasciculus; TPSC, pathway between the sensory thalamus and primary somatosensory cortex; AR, auditory radiation. * *p* < 0.05, ** *p* < 0.01, *** *p* < 0.001.

**Figure 3:**
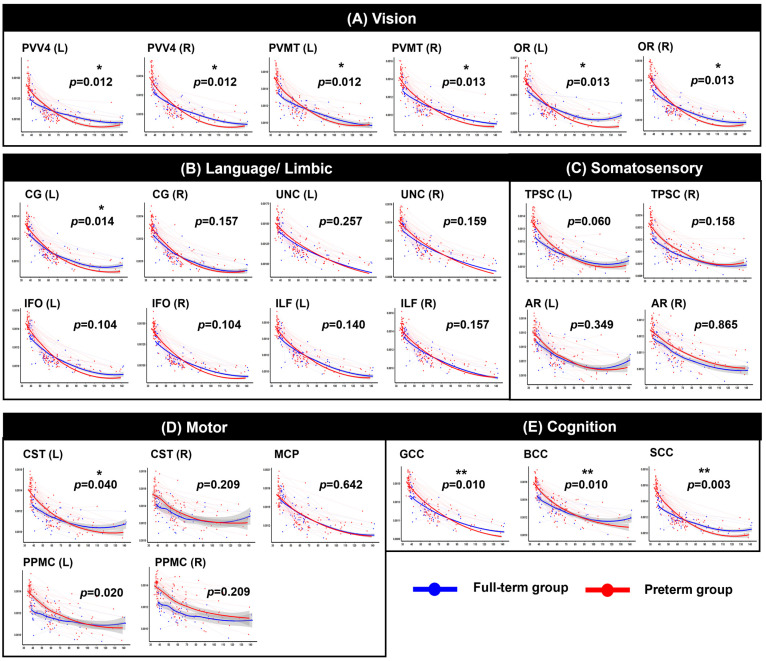
Longitudinal trajectories of mean diffusivity (MD) across PMA. Each circle represents an individual scan, and thin lines connect repeated measures from the same participant. The red color indicates the preterm group, and the blue color indicates the full-term group. The shaded gray area indicates the 95% confidence interval of the fitted curve. Abbreviations: CST, corticospinal tract; PPMC, pathway connecting the premotor and primary motor cortices; MCP, middle cerebellar peduncle; gCC, genu of the corpus callosum; sCC, splenium of the corpus callosum; PV–V4, pathway between the V1 (primary visual area)/V2 (secondary visual area) and V4; PV-MT, pathway connecting the V1/V2 and V5/MT (middle temporal visual area); OR, optic radiation; CG, cingulum; UNC, uncinate process; IFO, inferior fronto-occipital fasciculus; ILF, inferior longitudinal fasciculus; TPSC, pathway between the sensory thalamus and primary somatosensory cortex; AR, auditory radiation. * *p* < 0.05, ** *p* < 0.01.

**Figure 4:**
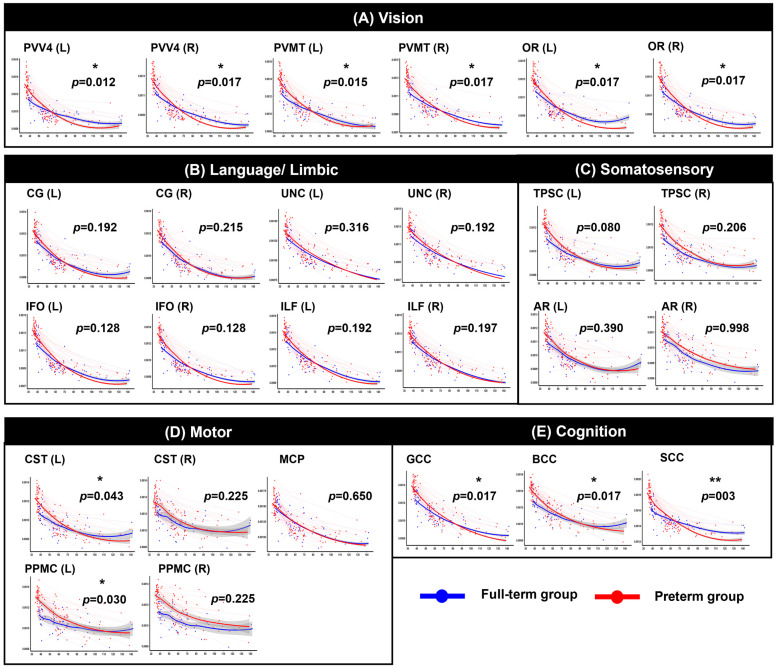
Longitudinal trajectories of radial diffusivity (RD) across PMA. Each circle represents an individual scan, and thin lines connect repeated measures from the same participant. The red color indicates the preterm group, and the blue color indicates the full-term group. The shaded gray area indicates the 95% confidence interval of the fitted curve. Abbreviations: CST, corticospinal tract; PPMC, pathway connecting the premotor and primary motor cortices; MCP, middle cerebellar peduncle; gCC, genu of the corpus callosum; sCC, splenium of the corpus callosum; PV–V4, pathway between the V1 (primary visual area)/V2 (secondary visual area) and V4; PV-MT, pathway connecting the V1/V2 and V5/MT (middle temporal visual area); OR, optic radiation; CG, cingulum; UNC, uncinate process; IFO, inferior fronto-occipital fasciculus; ILF, inferior longitudinal fasciculus; TPSC, pathway between the sensory thalamus and primary somatosensory cortex; AR, auditory radiation. * *p* < 0.05, ** *p* < 0.01.

**Figure 5:**
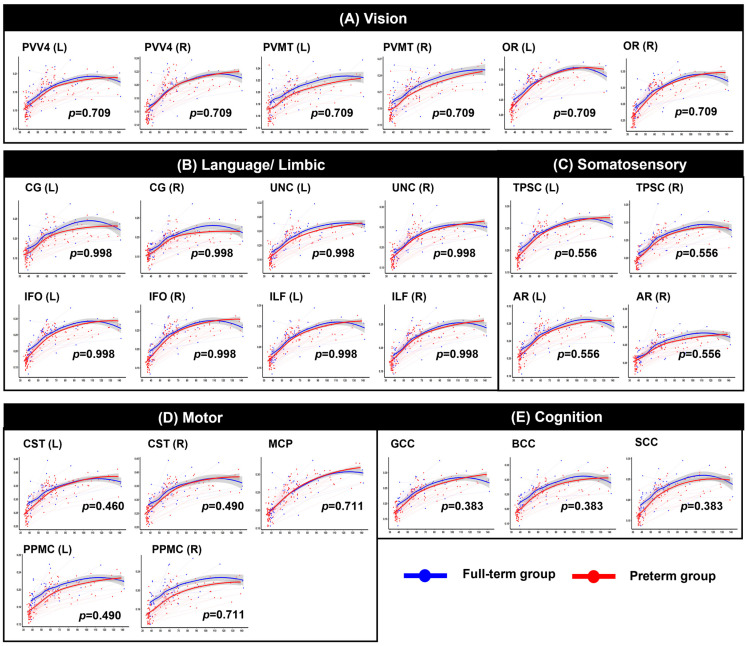
Longitudinal trajectories of fractional anisotropy (FA) across PMA. Each circle represents an individual scan, and thin lines connect repeated measures from the same participant. The red color indicates the preterm group, and the blue color indicates the full-term group. The shaded gray area indicates the 95% confidence interval of the fitted curve. Abbreviations: CST, corticospinal tract; PPMC, pathway connecting the premotor and primary motor cortices; MCP, middle cerebellar peduncle; gCC, genu of the corpus callosum; sCC, splenium of the corpus callosum; PV–V4, pathway between the V1 (primary visual area)/V2 (secondary visual area) and V4; PV-MT, pathway connecting the V1/V2 and V5/MT (middle temporal visual area); OR, optic radiation; CG, cingulum; UNC, uncinate process; IFO, inferior fronto-occipital fasciculus; ILF, inferior longitudinal fasciculus; TPSC, pathway between the sensory thalamus and primary somatosensory cortex; AR, auditory radiation.

After FDR correction within the functionally grouped WM regions, significant group-by-age interaction effects were identified across multiple tracts, indicating significantly altered trajectories between the two groups (see Figure 2, Figure 3, Figure 4 and Figure 5 and Supplementary Tables S1–S4 for detailed results). Motor-related pathways, including the left CST (*p* = 0.048 for AD, *p* = 0.040 for MD, *p* = 0.043 for RD) and left PPMC (*p* = 0.015 for AD, *p* = 0.020 for MD, *p* = 0.030 for RD), showed significant differences between the groups by age. Visual processing pathways also differed significantly between the two groups. In particular, the bilateral OR (left: *p* = 0.012, right: *p* = 0.012 for AD; left: *p* = 0.013, right: *p* = 0.013 for MD; left: *p* = 0.017, right: *p* = 0.017 for RD), PVV4 (left: *p* = 0.006, right: *p* = 0.012 for AD; left: *p* = 0.012, right: *p* = 0.012 for MD; left: *p* = 0.012, right: *p* = 0.017 for RD), and PVMT (left: *p* = 0.012, right: *p* = 0.012 for AD; left: *p* = 0.012, right: *p* = 0.013 for MD; left: *p* = 0.015, right: *p* = 0.017 for RD) demonstrated consistent alterations. In cognition-related regions, all subregions of the corpus callosum–the genu (*p* = 0.006 for AD, *p* = 0.010 for MD, *p* = 0.017 for RD), body (*p* = 0.007 for AD, *p* = 0.010 for MD, *p* = 0.017 for RD), and splenium (*p* < 0.001 for AD, *p* = 0.003 for MD and RD)–exhibited significant interaction effects across all three diffusivity metrics. Moreover, for the somatosensory system, the left TPSC showed a significant group-by-age interaction in AD (*p* = 0.036). Finally, in the limbic-language system, the left CG exhibited a significant interaction with MD (*p* = 0.014). In contrast, no significant group-by-age interactions were detected in FA across any of the examined tracts.

### 3.3. Inter-Regional Partial Correlations Based on Residuals from the Longitudinal Model

**Figure 6:**
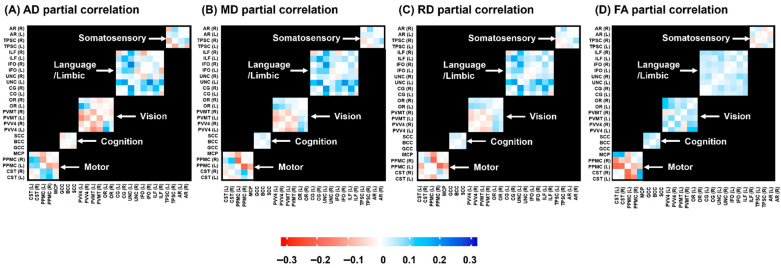
Group differences in inter-regional correlations of WM microstructural changes. WM tracts were functionally outlined into five domains: motor, cognitive, visual, language/limbic, and somatosensory. Red and blue colors indicate stronger correlations in the preterm and full-term groups, respectively, with color intensity indicating the magnitude of between-group differences. Abbreviations: CST, corticospinal tract; PPMC, pathway connecting the premotor and primary motor cortices; MCP, middle cerebellar peduncle; gCC, genu of the corpus callosum; sCC, splenium of the corpus callosum; PV–V4, pathway between the V1 (primary visual area)/V2 (secondary visual area) and V4; PV-MT, pathway connecting the V1/V2 and V5/MT (middle temporal visual area); OR, optic radiation; CG, cingulum; UNC, uncinate process; IFO, inferior fronto-occipital fasciculus; ILF, inferior longitudinal fasciculus; TPSC, pathway between the sensory thalamus and primary somatosensory cortex; AR, auditory radiation.

Based on the predefined functional classifications, we performed partial correlation analyses within functionally grouped WM regions, including the motor, cognitive, visual, language/limbic, and somatosensory subsets. We calculated the difference in the correlation matrix between the full-term and preterm groups (full-term group–preterm group) for each of the four DTI measures (AD, MD, RD, and FA). Figure 6 shows a 26 × 26 correlation heatmap, with positive values indicating a higher correlation in the full-term group than in the preterm group and negative values indicating a higher correlation in the preterm group than in the full-term group. Notably, the WM pathways associated with motor and visual functional pathways showed greater correlations with other tracts across all four metrics in the preterm group.

### 3.4. Associations Between Individual WM Developmental Slopes over the First 2 Years and Neurodevelopmental Outcomes in the Preterm Group

To understand the associations between individual differences in the rates of WM development slopes and neurodevelopmental outcomes in the preterm group over the first 2 years, we analyzed the partial correlations between the individual slopes of diffusion metrics (AD, MD, RD, and FA) in each WM tract and the BSID III scores. However, the statistical significance in cognition, language, and social-emotion across the BSID III scores disappeared after the FDR-corrected for multiple comparisons. The motor outcome was associated with many functional WM developmental slopes (Table 2). This suggests that multiple functional WM developmental changes during the first 2 years are most strongly associated with motor function scores and may serve as a fundamental mechanism underlying early neurodevelopment. Notably, the MCP in AD, MD, RD, and FA were significantly correlated with motor function (r = −0.397, *p* = 0.010 for AD; r = −0.420, *p* = 0.006 for MD; r = −0.430, *p* = 0.005 for RD; r = 0.432, *p* = 0.005 for FA). Table 2 presents the tracts that were significantly associated with neurodevelopmental outcomes.

## 4. Discussion

In this study, we showed that the preterm group exhibited an altered longitudinal microstructural trajectory in WM maturation of motor, visual, and cognition-related pathways in DTI measures compared to the full-term group over the first 2 years of life. These pathways exhibited greater inter-regional coherence within the motor- or visual-related pathways in the preterm group, reflecting a more advanced anatomical integration for developmental function. Furthermore, the motor outcome on the BSID-III examination at 2 years was most significantly associated with multiple functional WM pathways, highlighting the association between individual rates of WM change and neurodevelopmental outcomes in the preterm group.

Consistent with previous DTI studies [7,35], we found that the preterm group showed a general WM maturation pattern, such as a decrease in FA and an increase in AD, MD, and RD across 26 ROI WM tracts, compared to the full-term group during a few months of life [36]. These maturation patterns in the preterm group are closely related to immature axons and dendrites, which have not undergone the critical WM development processes that typically occur during the fetal period. Dubois et al. (2008) proposed a theoretical framework for preterm birth-related alterations in WM during the fetal and infant stages using WM indices [7]. In the initial stage, the fiber organization within the fascicles develops and becomes directionally organized, leading to an increase in AD and FA. During the intermediate stage, the proliferation of glial cells and cytoskeletons leads to increased cellular density within the WM, decreasing water content, and increasing membrane density, which is linked to an overall decline in AD, MD, and RD. In the final stage, oligodendrocyte ensheathment and macromolecule accumulation restrict water diffusion, leading to a further decrease in AD, MD, and RD, while FA increases. Furthermore, extensive extrauterine exposure after birth in the neonatal intensive care unit, such as pain, light, and noise, may increase vulnerability to early developmental abnormalities, further delaying myelination and WM maturation [37]. Although the preterm group initially exhibited delayed developmental milestones, they subsequently demonstrated pronounced alterations in motor, visual, and cognitive pathways across AD, MD, and RD during the first 2 years of life in the present study. Motor- and visual-related pathways, which are known to undergo the earliest and most rapid myelination [6,38], may be especially amenable to such compensatory remodeling, potentially contributing to the observed convergence of developmental trajectories between preterm and full-term infants. Notably, these functional pathways, such as the motor and visual pathways, also exhibited greater inter-regional coherence in the preterm group. These inter-regional coherences, capturing synchronized and pathway-specific maturation patterns, suggest that early immaturity in white matter development may be compensated for within specific functional pathways through compensatory mechanisms and experience-dependent neuroplasticity. The preterm infant brain is particularly notable for its plasticity because it develops at a rapid pace during this early critical point in development. Although compensatory mechanisms of brain plasticity can operate across multiple levels, including the physiological, metabolic, and environmental domains [23], the developing infant brain remains intrinsically vulnerable to environmental influences. Although extrauterine exposure can have both positive and negative effects, enriched experiences, such as neonatal handling and sensory stimulation, exert lasting effects on neurobehavioral development in preterm animal models and human infants by promoting structural changes via neurotrophic mechanisms [39,40]. These converging lines of evidence highlight that, although early brain development is a vulnerable period, it also presents a critical opportunity for targeted interventions. By providing supportive external influences during the first 2 years of life, it may be possible to enhance neuroplasticity, guide WM development along more typical pathways. However, increased inter-regional coherence observed in motor- and visual-related pathways not only interpreted as a compensatory mechanism; but also interpreted as an indicator of potential atypical reorganization in preterm infants, reflecting over-coupling of neural networks. França et al. (2024) reported that heightened brain synchronization within early-developing sensorimotor in preterm infants may indicate atypical reorganization or delayed network differentiation, rather than efficient maturation [41]. Although such coupling may support short-term functional convergence, caution is warranted in interpreting to long-term neurodevelopmental outcomes. Further research is needed to determine whether these inter-regional coherences are adaptive or compensatory and to identify optimal windows of brain plasticity for learning and intervention through the integration of early brain developmental measures with later neurodevelopmental assessments.

The majority of previous studies examining the relationship between WM maturation and functional outcomes for the identification of high-risk groups have relied on cross-sectional or group-level longitudinal analyses. However, to directly capture inter-individual variability and enable inference of individual developmental trajectories, analyzing individual rates of change may offer methodological and interpretative advantages beyond prior approaches. In the present study, we elucidate the relationship between longitudinal brain microstructural maturation and functional outcomes at the individual level, examining partial correlations between individual rates of change in AD, MD, RD, and FA for each tract over 2 years and neurodevelopmental outcomes in the preterm group. Among BSID-III scores, motor outcomes showed significant correlations with several functional white matter pathways, with FA consistently demonstrating significance across nearly all pathways. FA reflects a composite measure of white matter microstructural organization, integrating multiple underlying features such as axonal coherence, fiber density, myelination, and membrane integrity. In preterm infants, maturational changes in anisotropy are greater and exhibit a wider dynamic range than those of other diffusion measure [42]. These properties suggest that longitudinal FA change rates may represent the most sensitive indicator of microstructural maturation associated with motor development in preterm infants. Moreover, we found that the MCP emerged as a key tract that was significantly associated with motor function across all diffusion metrics [43,44,45], suggesting that a steeper rate of change in the MCP over the first two years was associated with better motor outcomes. Our findings revealed that the early microstructural development of the MCP, a major cerebellar afferent pathway connecting the cerebrum and cerebellum [46,47], may play a core role in shaping subsequent motor-related functions [43]. Schmahmann et al. (2010) emphasized that intact cerebellar connectivity is essential for both neuromotor and neurocognitive development in specific functional domains through reciprocal cerebrocerebellar circuits [48]. Our previous study showed that interhemispheric WM connections in the MCP were associated with a strong predictive accuracy for motor and cognitive impairment in preterm infants using multivariable linear regression [17]. Specifically, aberrant development of the MCP has been increasingly reported in individuals with developmental motor delay [49] language disorders [50] autism spectrum disorders [51,52], attention-deficit/hyperactivity disorder, and cognitive deficits [53,54]. Although the correlations with BSID-III motor scores observed in the present study were statistically significant, they should not be interpreted as evidence of direct causality. Neurodevelopment in preterm infants is inherently multifactorial, reflecting the complex interplay of biological and environmental influences, including inflammatory exposure, nutritional status, and the intensity of early sensory stimulation. Accordingly, the observed association between microstructural maturation of the MCP and subsequent motor outcomes should be interpreted within this broader clinical context, rather than as a direct mechanistic relationship.

Based on the findings of this study, early preterm birth induces region-specific neuroplasticity and compensates for the development of WM. These pathways are particularly pronounced in the regions involved in motor and visual functions, which are characterized by early structural maturation. Moreover, the identification of sensitive markers for the longitudinal rate of change in functionally distinct WM pathways offer valuable insights into the neurodevelopmental consequences of premature birth and highlights potential targets for early intervention. The present findings suggest that longitudinal trajectories of white matter maturation, rather than single time-point measures, may provide clinically meaningful information for the early identification of preterm infants at increased risk for later motor impairments. Although the present study does not establish direct causal relationships between WM maturation and neurodevelopmental outcomes, the observed maturation patterns may nonetheless serve as early prognostic markers when interpreted within a broader clinical context. Importantly, the first two years of life represent a critical period of heightened brain plasticity, during which deviations in white matter maturation trajectories may help inform the optimal timing of targeted early interventions. Infants exhibiting atypical or attenuated maturation slopes may therefore benefit from closer developmental monitoring and earlier, individualized neurodevelopmental support. Future studies integrating longitudinal neuroimaging trajectories with clinical, environmental, and biological factors are warranted to further refine early risk stratification strategies and to translate these findings into practical guidance for neonatal care.

However, this study has certain limitations that should be addressed in future studies. First, the population of the full-term group was smaller than that of the preterm group owing to the difficulty in enrolling neonates, although we included all eligible neonates in our hospital as much as possible. In addition, although sex was included as a covariate in the analyses, sex-specific differences in brain maturation trajectories and neurodevelopmental outcomes may not have been fully captured given the sample size. Future studies with larger and more balanced cohorts, as well as stratified or sex-specific analyses, will be important to more precisely delineate the effects of prematurity while accounting for potential influences of group size imbalance and sex-related developmental differences. Second, we only considered maternal age as an environmental factor and disregarded other factors, such as respiratory support, inflammation, infections, nutritional status, and the intensity of sensory stimulation, breasting status and mode of delivery, and the level of sensory stimulation during hospitalization. These factors have been shown to interact with neurodevelopmental trajectories and white matter microstructural maturation in preterm infants. The absence of these variables in the present analyses limits our ability to disentangle the relative contributions of intrinsic brain developmental processes versus extrinsic clinical and environmental influences. This may have compromised our assessment of the differential effects of WM development on the later BSID-III outcomes. Hence, future studies should consider a more balanced sample size and/or multicenter design and include various environmental factors to consolidate and improve the disentanglement of individual developmental trajectories. The early postnatal period represents a critical window during which ongoing brain maturation may remain amenable to intervention. Although these associations do not imply causality, linking early brain developmental trajectories with later motor outcomes may help inform the timing and targeting of early neurodevelopmental interventions.

## Figures and Tables

**Table 1:** Demographic and clinical characteristics of the full-term and preterm groups.

	Full-Term Group (*n* = 23; Scan = 52)	Preterm Group (*n* = 58; Scan = 122)	*p*-Value
Gestational age, weeks (SD)	38.72 (1.26)	29.33 (3.70)	**<0.001**
Age at first scan, weeks (SD)	42.48 (6.37)	38.86 (6.89)	**0.002**
Birth weight, g (SD)	3217.17 (394.09)	1320.28 (610.16)	**<0.001**
Male, n (%)	17 (70.8%)	24 (41.3%)	**0.017**
Maternal age (SD)	34.69 (4.12)	33.91 (3.74)	0.300
BSID-III			
Cognition	105.00 (17.56)	98.07 (13.55)	0.130
Language	97.26 (12.71)	90.02 (13.15)	**0.042**
Motor	108.84 (14.39)	97.71 (15.56)	**0.008**
Social emotion	100.53 (17.31)	100.36 (18.63)	0.973

**Table 2:** Significant correlation pathways (FDR-corrected *p* < 0.05) between individual rate of change and neurodevelopment outcomes in the preterm group.

Bayley Subset	Metrics	Function Related	Tract	Correlation r	*p*-Value	* FDR
Motor outcome	AD	Motor	MCP	−0.397	0.002	**0.010**
	MD	Motor	MCP	−0.420	0.001	**0.006**
		Limbic/Language	CG	−0.337	0.010	**0.031**
	RD	Motor	MCP	−0.430	0.001	**0.005**
		Cognition	GCC	−0.316	0.016	**0.045**
		Limbic/Language	CG	−0.373	0.004	**0.016**
			UNC	−0.332	0.011	**0.033**
		Somatosensory	TPSC	−0.341	0.009	**0.030**
	FA	Motor	MCP	0.432	0.001	**0.005**
			PPMC	0.364	0.005	**0.018**
			CST	0.384	0.003	**0.013**
		Cognition	GCC	0.431	0.001	**0.005**
			BCC	0.450	0.000	**0.005**
			SCC	0.469	0.000	**0.005**
		Vision	OR	0.446	0.000	**0.005**
			PVV4	0.363	0.005	**0.018**
		Limbic/Language	CG	0.427	0.001	**0.005**
			IFO	0.394	0.002	**0.010**
			ILF	0.451	0.000	**0.005**
		Somatosensory	TPSC	0.430	0.001	**0.005**
			AR	0.395	0.002	**0.010**

Abbreviations: AD, axial diffusivity; MD, mean diffusivity; RD, radial diffusivity; FA, fractional anisotropy; CST, corticospinal tract; Pre-primary MC, pathway connecting the premotor and primary motor cortices; MCP, middle cerebellar peduncle; GCC, genu of the corpus callosum; BCC, body of the corpus callosum; SCC, splenium of the corpus callosum; PV–V4, pathway between the V1 (primary visual area)/V2 (secondary visual area) and V4; PV-MT, pathway connecting the V1/V2 and V5/MT (middle temporal visual area); OR, optic radiation; CG, cingulum; UNC, uncinate process; IFO, inferior fronto-occipital fasciculus; ILF, inferior longitudinal fasciculus; TPSC, pathway between the sensory thalamus and primary somatosensory cortex; AR, auditory radiation. * Statistically significant at *p* < 0.05, for multiple comparisons by BSID 4 subscales.

## Data Availability

The datasets generated and/or analyzed during the current study are not publicly available because of the inability to share personal information according to research ethics but are available from the corresponding author upon reasonable request. Correspondence and requests for materials should be addressed to G.Y.L. (lgl6870@naver.com) and H.J.L. (blesslee77@hanmail.net).

## Supplementary Materials

The following supporting information can be downloaded at: https://www.mdpi.com/article/10.3390/jcm15020823/s1, Table S1. Linear mixed model results for the age-by-group interaction of axial diffusivity. Table S2. Linear mixed model results for the age-by-group interaction of mean diffusivity. Table S3. Linear mixed model results for the age-by-group interaction of radial diffusivity. Table S4. Linear mixed model results for the age-by-group interactions of fractional anisotropy.

## Abbreviations

The following abbreviations are used in this manuscript:

WM	White matter
DTI	Diffusion tensor imaging
LME	Linear mixed effect
GA	Gestational age
TEA	Term-equivalent age
BSID	Bayley Scales of Infant and Toddler Development
MRI	Magnetic resonance imaging
FA	Fractional anisotropy
MA	Mean diffusivity
AD	Axial diffusivity
RD	Radial diffusivity
CST	Corticospinal tract
PPMC	Pathway connecting the premotor and primary motor cortices
MCP	Middle cerebellar peduncle
GCC	Genu of the corpus callosum
BCC	Body of the corpus callosum
SCC	Splenium of the corpus callosum
PVV4	pathway between the V1/V2 and V4
PVMT	pathway connecting the V1/V2 and V5/MT
OR	Optic radiation
CG	Cingulum
UNC	Uncinate fasciculus
IFO	Inferior fronto-occipital fasciculus
ILF	Inferior longitudinal fasciculus
TPSC	Thalamo- primary somatosensory cortex
AR	Acoustic radiation

## References

[1] Bethlehem R.A.I., Seidlitz J., White S.R., Vogel J.W., Anderson K.M., Adamson C., Adler S., Alexopoulos G.S., Anagnostou E., Areces-Gonzalez A. (2022). Brain charts for the human lifespan. Nature.

[2] Gilmore J.H., Lin W., Prastawa M.W., Looney C.B., Vetsa Y.S., Knickmeyer R.C., Evans D.D., Smith J.K., Hamer R.M., Lieberman J.A. (2007). Regional gray matter growth, sexual dimorphism, and cerebral asymmetry in the neonatal brain. J. Neurosci..

[3] Knickmeyer R.C., Gouttard S., Kang C., Evans D., Wilber K., Smith J.K., Hamer R.M., Lin W., Gerig G., Gilmore J.H. (2008). A structural MRI study of human brain development from birth to 2 years. J. Neurosci..

[4] Li G., Nie J., Wang L., Shi F., Lyall A.E., Lin W., Gilmore J.H., Shen D. (2014). Mapping longitudinal hemispheric structural asymmetries of the human cerebral cortex from birth to 2 years of age. Cereb. Cortex.

[5] Lyall A.E., Shi F., Geng X., Woolson S., Li G., Wang L., Hamer R.M., Shen D., Gilmore J.H. (2015). Dynamic Development of Regional Cortical Thickness and Surface Area in Early Childhood. Cereb. Cortex.

[6] Dubois J., Dehaene-Lambertz G., Kulikova S., Poupon C., Hüppi P.S., Hertz-Pannier L. (2014). The early development of brain white matter: A review of imaging studies in fetuses, newborns and infants. Neuroscience.

[7] Dubois J., Dehaene-Lambertz G., Perrin M., Mangin J.F., Cointepas Y., Duchesnay E., Le Bihan D., Hertz-Pannier L. (2008). Asynchrony of the early maturation of white matter bundles in healthy infants: Quantitative landmarks revealed noninvasively by diffusion tensor imaging. Hum. Brain Mapp..

[8] Hüppi P.S., Dubois J. (2006). Diffusion tensor imaging of brain development. Semin. Fetal Neonatal Med..

[9] Filley C.M., Fields R.D. (2016). White matter and cognition: Making the connection. J. Neurophysiol..

[10] Kostovic I., Vasung L. (2009). Insights from in vitro fetal magnetic resonance imaging of cerebral development. Semin. Perinatol..

[11] Yap P.T., Fan Y., Chen Y., Gilmore J.H., Lin W., Shen D. (2011). Development trends of white matter connectivity in the first years of life. PLoS ONE.

[12] Ophelders D.R., Gussenhoven R., Klein L., Jellema R.K., Westerlaken R.J., Hütten M.C., Vermeulen J., Wassink G., Gunn A.J., Wolfs T.G. (2020). Preterm brain injury, antenatal triggers, and therapeutics: Timing is key. Cells.

[13] Pandit A., Robinson E., Aljabar P., Ball G., Gousias I., Wang Z., Hajnal J., Rueckert D., Counsell S., Montana G. (2014). Whole-brain mapping of structural connectivity in infants reveals altered connection strength associated with growth and preterm birth. Cereb. Cortex.

[14] Wang W., Wang M., Yang M., Zeng B., Qiu W., Ma Q., Jing X., Zhang Q., Wang B., Yin C. (2022). Transcriptome dynamics of hippocampal neurogenesis in macaques across the lifespan and aged humans. Cell Res..

[15] Jang Y.H., Ham J., Kasani P.H., Kim H., Lee J.Y., Lee G.Y., Han T.H., Kim B.-N., Lee H.J. (2024). Predicting 2-year neurodevelopmental outcomes in preterm infants using multimodal structural brain magnetic resonance imaging with local connectivity. Sci. Rep..

[16] Lean R.E., Han R.H., Smyser T.A., Kenley J.K., Shimony J.S., Rogers C.E., Limbrick D.D., Smyser C.D. (2019). Altered neonatal white and gray matter microstructure is associated with neurodevelopmental impairments in very preterm infants with high-grade brain injury. Pediatr. Res..

[17] Lee H.J., Kwon H., Kim J.I., Lee J.Y., Lee J.Y., Bang S., Lee J.-M. (2021). The cingulum in very preterm infants relates to language and social-emotional impairment at 2 years of term-equivalent age. NeuroImage Clin..

[18] Young J.M., Morgan B.R., Whyte H.E.A., Lee W., Smith M.L., Raybaud C., Shroff M.M., Sled J.G., Taylor M.J. (2017). Longitudinal Study of White Matter Development and Outcomes in Children Born Very Preterm. Cereb. Cortex.

[19] Geng X., Gouttard S., Sharma A., Gu H., Styner M., Lin W., Gerig G., Gilmore J.H. (2012). Quantitative tract-based white matter development from birth to age 2 years. NeuroImage.

[20] Kinney H.C., Brody B.A., Kloman A.S., Gilles F.H. (1988). Sequence of central nervous system myelination in human infancy: II. Patterns of myelination in autopsied infants. J. Neuropathol. Exp. Neurol..

[21] Paus T., Collins D., Evans A., Leonard G., Pike B., Zijdenbos A. (2001). Maturation of white matter in the human brain: A review of magnetic resonance studies. Brain Res. Bull..

[22] Guan Y., Wang Y., Zhou C., Wang J., Lu Y. (2025). Distinct patterns of cognitive enhancement: The role of motor experience in domain-specific and general cognitive functions. Neuroscience.

[23] Kolb B., Gibb R. (2011). Brain plasticity and behaviour in the developing brain. J. Can. Acad. Child Adolesc. Psychiatry.

[24] Volpe J.J. (2021). Primary neuronal dysmaturation in preterm brain: Important and likely modifiable. J. Neonatal Perinatal Med..

[25] Bastiani M., Cottaar M., Fitzgibbon S.P., Suri S., Alfaro-Almagro F., Sotiropoulos S.N., Jbabdi S., Andersson J.L. (2019). Automated quality control for within and between studies diffusion MRI data using a non-parametric framework for movement and distortion correction. NeuroImage.

[26] Jenkinson M., Beckmann C.F., Behrens T.E., Woolrich M.W., Smith S.M. (2012). FSL. NeuroImage.

[27] Smith S.M. (2002). Fast robust automated brain extraction. Hum. Brain Mapp..

[28] Tustison N.J., Avants B.B., Cook P.A., Zheng Y., Egan A., Yushkevich P.A., Gee J.C. (2010). N4ITK: Improved N3 bias correction. IEEE Trans. Med. Imaging.

[29] Ahn S.J., Lee J.Y., Lee J.Y., Lee Y.J., Lee J.M., Lee B.R., Kim J., Lee H.J. (2021). Brain White Matter Maturation and Early Developmental Outcomes in Preterm Infants With Retinopathy of Prematurity. Investig. Ophthalmol. Vis. Sci..

[30] Bunn A., Korpela M. (2020). An introduction to dplR. Process. Dplr.

[31] Montgomery D.C., Peck E.A., Vining G.G. (2021). Introduction to Linear Regression Analysis.

[32] Bates D., Mächler M., Bolker B., Walker S. (2015). Fitting linear mixed-effects models using lme4. J. Stat. Softw..

[33] Wood S.N. (2017). Generalized Additive Models: An Introduction with R.

[34] Shan G., Bayram E., Caldwell J.Z.K., Miller J.B., Shen J.J., Gerstenberger S. (2021). Partial correlation coefficient for a study with repeated measurements. Stat. Biopharm. Res..

[35] Akazawa K., Chang L., Yamakawa R., Hayama S., Buchthal S., Alicata D., Andres T., Castillo D., Oishi K., Skranes J. (2016). Probabilistic maps of the white matter tracts with known associated functions on the neonatal brain atlas: Application to evaluate longitudinal developmental trajectories in term-born and preterm-born infants. NeuroImage.

[36] Ment L.R., Kesler S., Vohr B., Katz K.H., Baumgartner H., Schneider K.C., Delancy S., Silbereis J., Duncan C.C., Constable R.T. (2009). Longitudinal brain volume changes in preterm and term control subjects during late childhood and adolescence. Pediatrics.

[37] Cheong J.L., Spittle A.J., Burnett A.C., Anderson P.J., Doyle L.W. (2020). Have outcomes following extremely preterm birth improved over time?. Semin. Fetal Neonatal Med..

[38] Richards J.E., Conte S., Lockman J.J., Tamis-LeMonda C.S. (2020). Brain development in infants: Structure and experience. The Cambridge Handbook of Infant Development: Brain, Behavior, and Cultural Context.

[39] DeMaster D., Bick J., Johnson U., Montroy J.J., Landry S., Duncan A.F. (2019). Nurturing the preterm infant brain: Leveraging neuroplasticity to improve neurobehavioral outcomes. Pediatr. Res..

[40] Fernández-Teruel A. (2022). The power of “touch” and early enriched stimulation: Neuroplasticity effects in rodents and preterm infants. Neural Regen. Res..

[41] França L.G.S., Ciarrusta J., Gale-Grant O., Fenn-Moltu S., Fitzgibbon S., Chew A., Falconer S., Dimitrova R., Cordero-Grande L., Price A.N. (2024). Neonatal brain dynamic functional connectivity in term and preterm infants and its association with early childhood neurodevelopment. Nat. Commun..

[42] Partridge S.C., Mukherjee P., Henry R.G., Miller S.P., Berman J.I., Jin H., Lu Y., Glenn O.A., Ferriero D.M., Barkovich A. (2004). Diffusion tensor imaging: Serial quantitation of white matter tract maturity in premature newborns. NeuroImage.

[43] Choi Y.H., Lee J.-M., Lee J.Y., Lee Y.-J., Ahn J.H., Lee H.J. (2021). Delayed Maturation of the Middle Cerebellar Peduncles at Near-Term Age Predicts Abnormal Neurodevelopment in Preterm Infants. Neonatology.

[44] Welniarz Q., Worbe Y., Gallea C. (2021). The Forward Model: A Unifying Theory for the Role of the Cerebellum in Motor Control and Sense of Agency. Front. Syst. Neurosci..

[45] Thomas A.R., Lacadie C., Vohr B., Ment L.R., Scheinost D. (2017). Fine Motor Skill Mediates Visual Memory Ability with Microstructural Neuro-correlates in Cerebellar Peduncles in Prematurely Born Adolescents. Cereb. Cortex.

[46] Travis K.E., Leitner Y., Feldman H.M., Ben-Shachar M. (2015). Cerebellar white matter pathways are associated with reading skills in children and adolescents. Hum. Brain Mapp..

[47] Habas C., Manto M. (2018). Probing the neuroanatomy of the cerebellum using tractography. Handb. Clin. Neurol..

[48] Schmahmann J.D. (2010). The role of the cerebellum in cognition and emotion: Personal reflections since 1982 on the dysmetria of thought hypothesis, and its historical evolution from theory to therapy. Neuropsychol. Rev..

[49] Kim D.Y., Park H.-K., Kim N.-S., Hwang S.-J., Lee H.J. (2016). Neonatal diffusion tensor brain imaging predicts later motor outcome in preterm neonates with white matter abnormalities. Ital. J. Pediatr..

[50] Asaridou S.S., Cler G.J., Wiedemann A., Krishnan S., Smith H.J., Willis H.E., Healy M.P., Watkins K.E. (2024). Microstructural Properties of the Cerebellar Peduncles in Children With Developmental Language Disorder. Neurobiol. Lang..

[51] Hanaie R., Mohri I., Kagitani-Shimono K., Tachibana M., Matsuzaki J., Hirata I., Nagatani F., Watanabe Y., Katayama T., Taniike M. (2018). Aberrant Cerebellar-Cerebral Functional Connectivity in Children and Adolescents With Autism Spectrum Disorder. Front. Hum. Neurosci..

[52] Sydnor L.M., Aldinger K.A. (2022). Structure, Function, and Genetics of the Cerebellum in Autism. J. Psychiatry Brain Sci..

[53] Lee J.C., Nopoulos P.C., Tomblin J.B. (2020). Procedural and declarative memory brain systems in developmental language disorder (DLD). Brain Lang..

[54] Stoodley C.J. (2014). Distinct regions of the cerebellum show gray matter decreases in autism, ADHD, and developmental dyslexia. Front. Syst. Neurosci..

