# Supplementary material for: Longitudinal White Matter Maturation in Preterm Infants: Functional Pathway-Specific Trajectories and Associations with Motor Outcomes
Source: J Clin Med. 2026 Jan 20;15(2):823. doi: 10.3390/jcm15020823 (PMC12842470; doi:10.3390/jcm15020823)
Supplement: Supplementary file 1 [file jcm-15-00823-s001.zip › jcm-4046037-supplementary.pdf]

Supplementary Table S1. Linear mixed model results for the age-by-group interaction of axial diffusivity.

Region		AD				
		Estimate	Std. Error	t-value	*p*-value	FDR
Motor	CST (L)	-1.230	0.512	-2.400	0.019	0.048
	CST (R)	-0.690	0.613	-1.126	0.264	0.330
	PPMC (L)	-1.419	0.450	-3.153	0.003	0.015
	PPMC (R)	-0.710	0.460	-1.543	0.127	0.212
	MCP	-0.443	0.886	-0.500	0.619	0.619
Cognition	GCC	-1.276	0.417	-3.064	0.004	0.006
	BCC	-1.321	0.470	-2.811	0.007	0.007
	SCC	-2.486	0.638	-3.897	0.000	<0.001
Vision	PVV4 (L)	-2.336	0.693	-3.370	0.001	0.006
	PVV4 (R)	-1.884	0.629	-2.996	0.004	0.012
	PVMT (L)	-1.933	0.711	-2.718	0.008	0.012
	PVMT (R)	-1.646	0.634	-2.597	0.012	0.012
	OR (L)	-1.499	0.557	-2.690	0.009	0.012
	OR (R)	-1.453	0.546	-2.662	0.010	0.012
Limbic/ Language	CG (L)	-1.009	0.449	-2.246	0.028	0.064
	CG (R)	-0.878	0.473	-1.857	0.069	0.107
	UNC (L)	-0.699	0.521	-1.342	0.181	0.181
	UNC (R)	-0.766	0.426	-1.799	0.080	0.107
	IFO (L)	-1.245	0.512	-2.433	0.018	0.064
	IFO (R)	-1.353	0.531	-2.547	0.014	0.064
	ILF (L)	-1.167	0.530	-2.203	0.032	0.064
	ILF (R)	-0.734	0.446	-1.646	0.106	0.121
Somatosensory	TPSC (L)	-1.218	0.456	-2.672	0.009	0.036
	TPSC (R)	-0.888	0.433	-2.049	0.045	0.090
	AR (L)	-0.541	0.442	-1.226	0.225	0.300
	AR (R)	-0.182	0.306	-0.594	0.555	0.555

Statistical significance was defined as FDR-corrected $p < 0.05$, correcting for multiple comparisons within functionally grouped WM regions, and is noted with *. Abbreviations: CST, corticospinal tract; PPMC, pathway connecting the premotor and primary motor cortices; MCP, middle cerebellar peduncle; gCC, genu of the corpus callosum; sCC, splenium of the corpus callosum; PV–V4, pathway between the V1 (primary visual area)/V2 (secondary visual area) and V4; PV-MT, pathway connecting the V1/V2 and V5/MT (middle temporal visual area); OR, optic radiation; CG, cingulum; UNC, uncinate process; IFO, inferior fronto-occipital fasciculus; ILF, inferior longitudinal fasciculus; TPSC, pathway between the sensory thalamus and primary somatosensory cortex; AR, auditory radiation

Supplementary Table S2. Linear mixed model results for the age-by-group interaction of mean diffusivity

Region		MD				
		Estimate	Std. Error	t-value	*p*-value	FDR
Motor	CST (L)	-1.387	0.561	-2.471	0.016	0.040
	CST (R)	-0.891	0.638	-1.396	0.167	0.209
	PPMC (L)	-1.351	0.458	-2.950	0.004	0.020
	PPMC (R)	-0.678	0.474	-1.430	0.158	0.209
	MCP	-0.397	0.850	-0.468	0.642	0.642
Cognition	GCC	-1.276	0.476	-2.677	0.010	0.010
	BCC	-1.361	0.505	-2.697	0.009	0.010
	SCC	-2.450	0.669	-3.661	0.001	0.003
Vision	PVV4 (L)	-2.173	0.671	-3.240	0.002	0.012
	PVV4 (R)	-1.711	0.605	-2.831	0.006	0.012
	PVMT (L)	-1.882	0.658	-2.861	0.006	0.012
	PVMT (R)	-1.500	0.584	-2.570	0.013	0.013
	OR (L)	-1.572	0.616	-2.553	0.013	0.013
	OR (R)	-1.554	0.594	-2.617	0.011	0.013
Limbic/ Language	CG (L)	-0.858	0.465	-1.847	0.070	0.014
	CG (R)	-0.743	0.493	-1.508	0.137	0.157
	UNC (L)	-0.615	0.540	-1.138	0.257	0.257
	UNC (R)	-0.740	0.436	-1.699	0.099	0.159
	IFO (L)	-1.316	0.577	-2.281	0.026	0.104
	IFO (R)	-1.438	0.583	-2.467	0.017	0.104
	ILF (L)	-1.070	0.545	-1.965	0.055	0.140
	ILF (R)	-0.705	0.457	-1.543	0.129	0.157
Somatosensory	TPSC (L)	-1.178	0.472	-2.494	0.015	0.060
	TPSC (R)	-0.833	0.468	-1.782	0.079	0.158
	AR (L)	-0.500	0.442	-1.131	0.262	0.349
	AR (R)	-0.054	0.317	-0.171	0.865	0.865

Statistical significance was defined as FDR-corrected $p < 0.05$, correcting for multiple comparisons within functionally grouped WM regions, and is noted with *. Abbreviations: CST, corticospinal tract; PPMC, pathway connecting the premotor and primary motor cortices; MCP, middle cerebellar peduncle; gCC, genu of the corpus callosum; sCC, splenium of the corpus callosum; PV–V4, pathway between the V1 (primary visual area)/V2 (secondary visual area) and V4; PV-MT, pathway connecting the V1/V2 and V5/MT (middle temporal visual area); OR, optic radiation; CG, cingulum; UNC, uncinate process; IFO, inferior fronto-occipital fasciculus; ILF, inferior longitudinal fasciculus; TPSC, pathway between the sensory thalamus and primary somatosensory cortex; AR, auditory radiation

Supplementary Table S3. Linear mixed model results for the age-by-group interaction of radial diffusivity

Region		RD				
		Estimate	Std. Error	t-value	*p*-value	FDR
Motor	CST (L)	-1.462	0.594	-2.461	0.017	0.043
	CST (R)	-0.995	0.658	-1.513	0.135	0.225
	PPMC (L)	-1.326	0.471	-2.813	0.006	0.030
	PPMC (R)	-0.656	0.485	-1.354	0.180	0.225
	MCP	-0.380	0.833	-0.456	0.650	0.650
Cognition	GCC	-1.265	0.513	-2.465	0.017	0.017
	BCC	-1.389	0.535	-2.595	0.012	0.017
	SCC	-2.415	0.690	-3.498	0.001	0.003
Vision	PVV4 (L)	-2.084	0.664	-3.139	0.002	0.012
	PVV4 (R)	-1.625	0.597	-2.721	0.009	0.017
	PVMT (L)	-1.852	0.640	-2.893	0.005	0.015
	PVMT (R)	-1.418	0.566	-2.506	0.015	0.017
	OR (L)	-1.603	0.654	-2.450	0.017	0.017
	OR (R)	-1.589	0.624	-2.549	0.014	0.017
Limbic/ Language	CG (L)	-0.779	0.478	-1.629	0.108	0.192
	CG (R)	-0.675	0.507	-1.330	0.188	0.215
	UNC (L)	-0.565	0.560	-1.009	0.316	0.316
	UNC (R)	-0.725	0.455	-1.594	0.120	0.192
	IFO (L)	-1.351	0.615	-2.196	0.032	0.128
	IFO (R)	-1.482	0.614	-2.414	0.019	0.128
	ILF (L)	-1.017	0.559	-1.819	0.075	0.192
	ILF (R)	-0.689	0.470	-1.467	0.148	0.197
Somatosensory	TPSC (L)	-1.154	0.486	-2.376	0.020	0.080
	TPSC (R)	-0.810	0.490	-1.654	0.103	0.206
	AR (L)	-0.478	0.451	-1.060	0.293	0.390
	AR (R)	0.001	0.331	0.002	0.998	0.998

Statistical significance was defined as FDR-corrected $p < 0.05$, correcting for multiple comparisons within functionally grouped WM regions, and is noted with *. Abbreviations: CST, corticospinal tract; PPMC, pathway connecting the premotor and primary motor cortices; MCP, middle cerebellar peduncle; gCC, genu of the corpus callosum; sCC, splenium of the corpus callosum; PV–V4, pathway between the V1 (primary visual area)/V2 (secondary visual area) and V4; PV-MT, pathway connecting the V1/V2 and V5/MT (middle temporal visual area); OR, optic radiation; CG, cingulum; UNC, uncinate process; IFO, inferior fronto-occipital fasciculus; ILF, inferior longitudinal fasciculus; TPSC, pathway between the sensory thalamus and primary somatosensory cortex; AR, auditory radiation

Supplementary Table S4. Linear mixed model results for the age-by-group interactions of fractional anisotropy

Region		FA				
		Estimate	Std. Error	t-value	*p*-value	FDR
Motor	CST (L)	294.049	171.761	1.712	0.092	0.460
	CST (R)	199.364	164.364	1.213	0.230	0.490
	PPMC (L)	112.768	106.382	1.060	0.294	0.490
	PPMC (R)	43.713	105.530	0.414	0.681	0.711
	MCP	47.363	127.390	0.372	0.711	0.711
Cognition	GCC	113.530	129.210	0.879	0.383	0.383
	BCC	150.206	144.930	1.036	0.304	0.383
	SCC	126.819	123.500	1.027	0.308	0.383
Vision	PVV4 (L)	34.792	92.684	0.375	0.709	0.709
	PVV4 (R)	35.220	86.840	0.406	0.687	0.709
	PVMT (L)	48.503	89.297	0.543	0.589	0.709
	PVMT (R)	40.583	90.220	0.450	0.655	0.709
	OR (L)	235.016	176.767	1.330	0.188	0.709
	OR (R)	177.189	154.696	1.145	0.256	0.709
Limbic/ Language	CG (L)	-7.723	112.738	-0.069	0.946	0.998
	CG (R)	-19.234	113.502	-0.169	0.866	0.998
	UNC (L)	-0.251	130.455	-0.002	0.998	0.998
	UNC (R)	37.231	120.799	0.308	0.759	0.998
	IFO (L)	170.586	152.625	1.118	0.268	0.998
	IFO (R)	184.610	140.445	1.314	0.193	0.998
	ILF (L)	38.382	112.503	0.341	0.734	0.998
	ILF (R)	59.380	101.656	0.584	0.561	0.998
Somatosensory	TPSC (L)	121.463	112.998	1.075	0.286	0.556
	TPSC (R)	78.885	133.131	0.593	0.556	0.556
	AR (L)	83.085	116.943	0.710	0.480	0.556
	AR (R)	-65.603	110.831	-0.592	0.556	0.556

Statistical significance was defined as FDR-corrected $p < 0.05$, correcting for multiple comparisons within functionally grouped WM regions, and is noted with *. Abbreviations: CST, corticospinal tract; PPMC, pathway connecting the premotor and primary motor cortices; MCP, middle cerebellar peduncle; gCC, genu of the corpus callosum; sCC, splenium of the corpus callosum; PV–V4, pathway between the V1 (primary visual area)/V2 (secondary visual area) and V4; PV-MT, pathway connecting the V1/V2 and V5/MT (middle temporal visual area); OR, optic radiation; CG, cingulum; UNC, uncinate process; IFO, inferior fronto-occipital fasciculus; ILF, inferior longitudinal fasciculus; TPSC, pathway between the sensory thalamus and primary somatosensory cortex; AR, auditory radiation
